# Antiphospholipid Antibodies and Lipids in Hematological Malignancies

**DOI:** 10.3390/ijms23084151

**Published:** 2022-04-08

**Authors:** Sonia Guadalupe Barreno-Rocha, Sandra Guzmán-Silahua, Sinaí-del-Carmen Rodríguez-Dávila, Guadalupe Estela Gavilanez-Chávez, Ernesto Germán Cardona-Muñoz, Carlos Riebeling-Navarro, Benjamín Rubio-Jurado, Arnulfo Hernán Nava-Zavala

**Affiliations:** 1 Unidad de Investigación Epidemiológica y en Servicios de Salud, CMNO OOAD Jalisco Instituto Mexicano del Seguro Social, Guadalajara 44340, Mexico; dra.barrenorocha@gmail.com (S.G.B.-R.); guzmansilahuasandra@gmail.com (S.G.-S.); sin.aayrdz@hotmail.com (S.-d.-C.R.-D.); g_gavilanez@hotmail.com (G.E.G.-C.); 2 Programa de Doctorado en Farmacología, Departamento de Fisiología, Centro Universitario Ciencias de la Salud, Universidad de Guadalajara, Guadalajara 44340, Mexico; german.cardona@academicos.udg.mx; 3 Unidad de Investigación en Epidemiologia Clínica, UMAE HP CMN-SXXI, Ciudad de México 06720, Mexico; criebnava@yahoo.com.mx; 4 Departamento Clínico de Hematología, División Onco-Hematologia, UMAE, Hospital de Especialidades, Centro Médico Nacional de Occidente, Instituto Mexicano del Seguro Social, Guadalajara 44340, Mexico; 5 Programa Internacional de Medicina, Universidad Autónoma de Guadalajara, Guadalajara 44670, Mexico; 6 Departamento de Inmunología y Reumatología del Hospital General de Occidente, Secretaría de Salud Jalisco, Guadalajara 45070, Mexico

**Keywords:** anti-β2 glycoprotein I, anticardiolipin, antiphospholipid antibodies, hematological malignancies, lipid, lupus anticoagulant

## Abstract

One of the main groups of lipids is phospholipids, which are mainly involved in forming cell membranes. Neoplastic processes such as cell replication have increased lipid synthesis, making tumor cells dependent on this synthesis to maintain their requirements. Antiphospholipid antibodies attack phospholipids in the cell membranes. Three main types of antiphospholipid antibodies are recognized: anti-β2 glycoprotein I (anti-β2GP-I), anticardiolipin (aCL), and lupus anticoagulant (LA). These types of antibodies have been proven to be present in hematological neoplasms, particularly in LH and NHL. This review on antiphospholipid antibodies in hematological neoplasms describes their clinical relationship as future implications at the prognostic level for survival and even treatment.

## 1. Overview of Lipids

Lipids are a group of organic compounds that are insoluble in water but soluble in organic solvents. This characteristic extends to several molecules such as fatty acids, phospholipids, sterols, sphingolipids, and terpenes [1]. Chemically, among the differences between these groups is the number of carbon atoms and double bonds and their positioning [2].

It is recognized that phospholipids form an essential part of the cell membrane and play a crucial role in different metabolic pathways and cellular functions; they act as sources of energy storage in specific processes and participate as signaling molecules and protein recruitment platforms [3].

The lipid bilayer is mainly formed by glycerophospholipids, sphingolipids, and sterols (mainly in mammals) [3]. Glycerophospholipids, cholesterol, and sphingomyelin are the predominant lipids in the cell membrane of animal cells. Covering lipids’ functional issues, they highlight their regulatory usefulness and the fact that they provide structure to this bilayer. Although not frequently included, other essential lipids are lipopolysaccharides and cardiolipins, the latter forming the mitochondria’s internal membrane [4].

### 1.1. Classification of Lipids

Referring to lipids, their structural and functional diversity has led to their significant classification. Sources such as The Lipid Library and Cyberlipids divide lipids into complex and simple according to the entities they produce when hydrolyzed. The simple lipids are produced with a maximum of two types of entities, such as acylglycerols by the hydrolysis of fatty acids and glycerol, while complex lipids such as glycerophospholipids are produced by hydrolysis involving fatty acids, glycerol, and a major group. This major group expands lipids’ diversity by defining phosphatidic acid, phosphatidylcholine (PC), phosphatidylserine, phosphatidylglycerol, phosphatidylinositol, and cardiolipin [5]. The LipidBank recognizes a third group, derived lipids, referring to those produced by simple lipids’ hydrolysis as alcohols and fatty acids [1].

Eukaryotic cell lipids contain chains of up to 26 carbons and multiple bonds, explaining the great diversity of glycerol-based lipids and their broad classification. Other sources classify the lipids related to their diversity: chemical diversity that confers specific properties of the lipids and diversity according to their composition that affects the lipids’ behavior on the membranes, depending on the interactions lipid–lipid or lipid–protein [3].

In 2001, Kishimoto first introduced the term “lipidomics” to describe lipid profiles and cell metabolism pathways. This area of lipid research, along with “metabolomics,” allows the finding of lipid biomarkers and metabolic patterns to characterize different diseases in a more specific way [6].

The LIPID MAPS is a classification system based on two essential compounds that derive many lipids: ketoacil and isoprene [1]; together, these two essential compounds summarize eight categories (Table 1) [6]:

### 1.2. Lipid Biosynthesis

All lipids that come from acetyl-CoA involve two pathways. The first one involves condensing acyl-carrying proteins derived from malonyl-Co and acetyl-CoA and a carbenium intermediate. This pathway produces acyl lipid chains such as fatty acids, phospholipids, and glycerolipids. The second pathway produces a condensation of pyrophosphate intermediates and carbocation; this route is a source of prenols, sterols, glycerophospholipids, and sphingolipids [7].

Glycerophospholipids, also called phospholipids, constitute 75% of the cells that make up the cell lipid membrane [5]. Phosphatidylcholine is one of the most abundant components in the membrane [6]. These contain esterified fatty acids in two positions: sn-1 and sn-2. Hydrolysis of the sn-2 ester has been carried out, which releases polyunsaturated fatty acids, and they are the substrate for multiple oxygenases. The cyclooxygenases catalyze double oxygenation to form arachidonic acid and prostaglandin endoperoxides. These bind to the membrane G-protein and carry out actions that contribute to the inflammatory response, such as prostaglandin products or thromboxanes that exert these receptors.

On the other hand, 5-lipooxygenase is able to convert arachidonic acid into leukotriene A4, which is hydrolyzed to leukotriene B4, or, if it is conjugated with glutathione, it produces C4; in this way, phospholipids can generate cellular signals in two ways: through the interaction of specific proteins and the alteration of the acyl lipid chain [7]. Besides contributing to the inflammatory response, these products also have a role in the allergic hypersensitivity response [7].

Other lipids belonging to this group, such as phosphatidylethanolamine, glycerophosphate, and cardiolipin, have a small polar head and a large hydrophobic tail, known as inverted cone lipids. A negative lipid curved cell layer forms if these lipids group together. This curve’s maintenance is vital for organelle formation, protein assembly, vesicle transport, and membrane fusion functions [6].

Phospholipases and lipid kinases act as second messengers; these hydrolyze a lipid substrate or, through phosphodiesterases, produce bioactive lipids. The enzymes involved in this process contribute to inflammation, oxidative stress, and infectious diseases. The phospholipases, enzymes that hydrolyze ester bonds of the phospholipids, have several members, such as phospholipase D, that produce phosphatidic acid and choline: phospholipase C, which converts phosphatidylinositol 4,5-bisphosphate into inositol 1,4,5, trisphosphate and diacylglycerol [5]; phospholipase A2, which catalyzes the hydrolysis of sn-2 phospholipids and produces arachidonic acid as a precursor to eicosanoids [7].

Another important group of the cell wall is the sphingolipids, whose great diversity is due to their length and type of sphingolipid base, their N-acyl chain, and their leading group. This category acts as a code to determine the functions of the lipid–protein interaction in the membrane. This kind of lipids consists of an aliphatic amino alcohol structure, being the ceramide and a central metabolite and precursor in synthesizing the sphingolipids [6].

Sterols can be localized in the cell wall, being, in cholesterol, the most important sterol in mammals [5]. Cholesterol comes from an endogenous way synthesized by most cells; however, its leading production site is in the hepatocytes and enterocytes, and it can be obtained through diet, but it does not exceed the endogenous production. Cholesterol is essential for cellular functions; it helps maintain the cell membrane’s permeability and fluidity. It also has an essential role in modulating transmembrane signaling pathways, and it is part of the composition of bile acids, vitamin D, and multiple steroid hormones. Maintaining the balance of cholesterol in the organism is of great importance as, otherwise, the lack or excess can be very harmful. Elevated cholesterol levels represent a risk factor in presenting different diseases, mainly in the development of cardiovascular disorders [3,8].

Knowing the composition of lipids and their action on cell functions comes from discovering genetic diseases related to lipids. These diseases result from mutations in different specialized signaling pathways by the enzymes involved in lipid metabolism. Some of these alterations in the homeostasis of the different lipids and their derivatives are reviewed below.

## 2. Overview of Phospholipids

Phospholipids are the main component of all cells and are involved in many biological processes. Their properties allow the formation of a lipid bilayer that gives the cell an external membrane that protects the intracellular environment from the extracellular environment [9].

Phospholipids are hydrocarbon chains joined by non-covalent bonds with hydrophobic properties. The lipid bilayer directs its hydrophobic components towards the cell membrane’s interior, while the hydrophilic components will perish on the external surface of the membrane. This organization allows a low permeability for solutes, such as nutrients and ions, besides providing a matrix where integral membrane proteins can be incorporated [9].

Lipids are part of essential cellular functions, mainly as a membrane barrier and matrix form in the protein functions, such as: DNA replication, cell division, solutes transport, protein translocation, and signal translation, among others [9].

In cells, phospholipids’ synthesis occurs in the endoplasmic reticulum, comprising more than 60% phospholipids. A large part of the phospholipid synthesis is transported to other organelles within the cell and even exported to the extracellular medium by specialized hepatocytes. For this reason, it is indispensable that the biosynthesis of phospholipids is sufficient to meet the previously mentioned demands [10].

Phosphatidylcholine (PC), the most abundant phospholipid in eukaryotic cell membranes, is also a component of bile, lung surfactant, and lipoproteins. Two pathways can carry out the biosynthesis of PC: the cytidine choline diphosphate (CDP) pathway (the Kennedy pathway), which is responsible for the major synthesis of PC; and phosphatidylethanolamine N-methyltransferase (PEMT). CDP-choline synthesizes 70% of liver CP [10,11,12]. Methylation by the PEMT is a reaction only carried out by hepatocytes and differentiated adipocytes [13].

## 3. Lipids and Their Relationship with Hematological Malignancies’ Biology

As described above, lipids exert multiple functions in the body’s healthy cells, such as signaling and energy storage; additionally, lipids are a required component in cell membranes’ structure to maintain optimal cell function. Thus, lipid synthesis converts nutrient-derived carbons into fatty acids, cholesterol, phosphoglycerides, eicosanoids, and sphingolipids [14].

Many cellular and membrane characteristics are a consequence of lipids’ properties, such as compositional complexity, versatility, fluidity, and lipid asymmetry. Their modification or a change in the concentration can affect the biomembranes, topology, spatial organization, and cellular functioning [14,15].

According to several observations, when there are an abnormal growth and proliferation of cells, as in cancer, there are multiple changes in metabolism, among them variations in mitochondrial respiration, and an altered lipid biosynthesis; because of its accelerated proliferation, neoplastic cells have high demands on their metabolism [14,16].

Thus, neoplastic cells show an increase in lipid synthesis. This lipid saturation protects these cells from oxidative damage by mitigating oxidative degradation [14,15].

Lipids also play a role in signaling molecules in cancer, acting as second messengers [14] for activating growth factor receptors and G-protein coupled receptors that activate cell migration, proliferation, and survival, as do phosphoinositides lysophosphatidic acids, respectively. Furthermore, they associate with autophagy, allowing neoplastic cells to conserve their energy during periods of nutritional limitation, thus promoting cell survival [14,15].

The vital importance of lipids in the membrane structure and cell signaling is evident, and their role in neoplastic cells regulates their survival and proliferation [14].

### Lipids, Phospholipids, and Hematological Malignancies

The different types of lipids are divided based on their composition and function; however, both physiological and pathological mechanisms participate in different types of neoplasms, both solid and hematological. One of the most studied enzymes is fatty acid synthase or acetyl-CoA carboxylase (ACC) in cancer genesis, especially in its role in maintaining membrane lipids and phospholipids.

Tumor cells depend on the action of fatty acid synthase (FAS) for de novo fatty acid synthesis for cell membrane genesis [17]. The function of FAS is to catalyze palmitate synthesis by condensing one acetyl-CoA molecule and seven malonyl-CoA molecules [18].

FAS is usually localized at most cells’ cytosol and less frequently at the cell membrane’s compartments, and the tyrosine-kinase type cell membrane receptor may phosphorylate and activate it. FAS provides additional lipids for the cell and conditions the structure and function for cell growth and survival [18].

FAS expression is relatively low in physiological conditions since its function is unnecessary because we have the required contribution of fatty acids in the diet [18]. On the contrary, fatty acid synthase overexpresses in different types of epithelial cancers, and in the same way, in hematological neoplasms such as leukemias, multiple myeloma, and diffuse B-cell non-Hodgkin’s lymphoma [18,19].

The malignant cells are dependent on hyperactive lipogenesis to maintain the high rate required to produce cell membranes during cell replication [17].

The morphology and nature of the tumor cell membrane change by the action of growth factors, second messengers, and domains, all of which usually respond to signals that prolong cell life. Cells with membranes densely composed of phospholipids and saturated fatty acids secondary to fatty acid synthase’s increased activity give tumor cells increased resistance to chemotherapeutic agents. Multiple authors are currently investigating selective ways to inhibit this enzyme activity as it may cause tumor cell death while respecting non-tumor cells; this is because of the observed dependence of part of the tumor cells on fatty acid synthase for the maintenance of the cell membrane [20].

Another critical enzyme responsible for lipid metabolism is acetyl-CoA carboxylase (ACC). It is an enzyme that converts acetyl-CoA to malonyl-CoA, and there are two types: ACC 1 and ACC 2. The most important type in the metabolism of lipids is ACC1, which can be found in the cytoplasm and is responsible for producing fatty acids. ACC2 is responsible for preventing the oxidation of fatty acids. These two enzymes have an essential role in many cancers because they help maintain cancer cells. The AMP-activated kinase (AMPK) is the ACC’s central regulatory kinase, inactivating ACC1 and ACC2 by phosphorylation. When the cells are in a favorable nutritional state, AMPK can be suppressed so that it does not exert its inhibitory action on ACC1 and ACC2, favoring higher production of fatty acids with higher energy and nutrients. Moreover, in different cancer phenotypes, it has been observed that there is an over-expression of ACC1 and ACC2 that lacks the domain that allows its hydroxylation, so it will help to prolong the life of the malignant cell by avoiding the oxidation of lipids and phospholipids [19].

As observed, FAS and ACC and their enzymatic activity represent an essential pillar in the physiopathology of multiple neoplasms, and they have been, lately, the focus of many researchers to develop new anticancer therapies. At the same time, the knowledge arose that several neoplasms positively express these enzymes, mostly FAS. Among the most remarkable solid tumors are breast, prostate, colon, ovarian, thyroid, lung, and stomach cancers; within the hematological neoplasms studied so far are diffuse large B-cell non-Hodgkin lymphoma, leukemias, multiple myeloma, and mantle lymphoma. However, in hematological neoplasms, these alterations in the metabolism of fatty acids, lipids, and phospholipids with their other derivatives have not been studied so extensively. Moreover, the relevance of FAS and ACC in hematological neoplasms is not fully understood [17,21].

## 4. Phospholipids as a Target of Antibodies in the Immune Response

### 4.1. Overview of Antiphospholipid Antibodies

These antibodies are directly against anionic phospholipids or protein–phospholipid complexes in the cell membrane [22,23]. Three main types of antibodies are classified within this heterogenic group: anti-β2 glycoprotein I (anti-β2GP-I), anticardiolipin (aCL), and lupus anticoagulant (LA) [22,24,25,26]. All of them are responsible for the wide range of clinical manifestations of antiphospholipid syndrome [23,26].

One of these antibodies’ main characteristics is that they are not specific for antiphospholipid syndrome (APS) since they can be found in healthy individuals, persons with a history of thrombosis, morbidity in pregnancy, or patients with autoimmune pathologies [27].

### 4.2. Anti-β2 Glycoprotein I (anti-β2GP-I)

Β2 glycoprotein I is a 50 kDa glycoprotein with 5 “sushi” domains (in reference to the form that takes); the first domains hold the binding site for most anti-β2GP-I and the last domain for anionic phospholipids. The conformational changes allow the exposure of these protein domains for antibody binding: the glycoprotein exists in a closed circular conformation in plasma and an open hook-like form with a C-terminal domain. Pathological anti-β2GP-I antibodies recognize the epitope in the N-terminal domain I that is exposed in the last bound to the surface [24,25,28,29]. These antibodies are known activators of endothelial cells, monocytes, neutrophils, and platelets, using pathways such as annexin A2 involving Toll type 4, type 2, and type 7 receptors (TLR4, TLR2, and TLR7) in lipid rafts [28,29]. They are currently considered the critical antibody in developing the pathogenesis of APS [28]. Furthermore, for being considered a positive test in anti-β2GP-I, it is necessary to have two or more positive plasma measurements with a difference of 12 weeks between each sample [22].

### 4.3. Anticardiolipin Antibodies (aCL)

Cardiolipin is an anionic type of phospholipid located mainly in the inner mitochondrial membrane of all cells, helping maintain the mitochondrial membrane’s fluidity and regulating the electron transport chain and programmed cell death [30]. For the antibodies that attack cardiolipin, the main target is the electronegative phospholipid associations, while their specificity will depend on the type (IgG or IgM) and the titers of the antibodies. Positivity is accepted by detecting these antibodies’ levels at medium or high titers, either in serum or plasma, on two or more occasions, with at least 12 weeks of difference between the samples [22].

### 4.4. Lupus Anticoagulant (LA)

LAs are known immunoglobulins that can activate phospholipid-dependent coagulation reactions, and they are helpful predictors of fetal morbidity [31]. These antibodies are most related to thrombotic events in APS [25]. To consider that the test is positive, the LA should be found in plasma on two or more occasions with different intakes more than 12 weeks apart [22].

The presence of antiphospholipid antibodies (aPLs) in malignant neoplasms has been demonstrated continuously, as well as the association of cancer with thrombosis [23]; this allows us to establish a connection between antibodies and cancer [22].

The tumor cell coagulation system interacts with platelets and the fibrinolytic system to generate thrombin, activating the coagulation cascade. Simultaneously, fibrin and the tissue factor can be mediators of fibrin deposition and platelet activation [22,26], and these exact mechanisms participate in the aPL-related thrombosis in APS [26]. In addition to this, some pathophysiological mechanisms for the association of aPL with cancer are (a) the production of autoantibodies in the presence of tumor antigens; (b) the secretion of aPL by tumor cells; (c) the production of immunoglobulins with aPL activities [26]. In patients with various neoplasms, their positive aPLs ranged from 20 to 60% [23].

## 5. Autoantibodies in Hematological Malignancies

B lymphocytes are responsible for producing antibodies, which are proteins that help modulate and activate the immune system, recognize antigens or proteins from foreign microorganisms, and lead to the activation of the inflammation cascade [32].

In patients with cancer, the frequency of displaying positive tests and increased titers of antiphospholipid antibodies (aPLs), such as aCL, AL, and anti β2 GP I, has been studied on multiple occasions [22,23,26,33,34]; it has been proven that the risk of presenting an elevation of these antibodies is predominant in solid tumors [33].

In addition to aPLs, other antibodies have manifested themselves with neoplasms. One of them is the anti-cytoplasmic neutrophil antibodies (ANCAs) [35]; associations of these antibodies’ expression with cancer may coexist with a systemic and localized inflammatory response [36]. Additionally, the other antibodies’ are the antinuclear antibodies (ANAs); they have been detected in multiple neoplasms [37,38,39], both hematological [40,41,42], in pulmonary [37,38], colorectal [37], or breast cancer, with high prevalence in these types of cancer [39].

In Table 2, we present a summary of the presence of the diverse types of antibodies on the hematological neoplasms:

### 5.1. aPL

Since the 1990s, there have been different studies of patients where aPL is associated with hematological malignancies, particularly Hodgkin’s lymphoma (HL), NHL, and acute leukemia [22].

There are studies where up to a third of patients with hematological malignancies are aPL-positive and studies where 100% of the cancer patients were up to 24% aPL-positive [26]. In a study in 2002, Genvresse et al. [44] published an aPL positivity of up to 27% of their total population of NHL patients [44]. In 2014, Uskudar et al. [43] carried out a transversal study, screening 54 patients with HL and NHL for antiphospholipid antibodies and finding a prevalence in 38% of the patients with HL and 62% in the NHL patients [43]. Pusterla y col. [45] reported that almost 30% of patients with lymphoma were aPL-positive, and the presence of thrombosis was higher in patients with lymphoma than in those without lymphoma [45,46].

### 5.2. ANCA

It is known that ANCAs attack proteins located in the neutrophil granules [35]. In order to stop and kill a variety of microorganisms, neutrophils create an extracellular structure of proteins, DNA, and various components, called extracellular neutrophil traps (NETs), and a malfunction of these structures can lead to the triggering of immune-mediated pathogenesis and even lead to immune diseases [47]. Recently, these NETs have been recognized as inducers of ANCA production, relating the production mechanism with the chronic inflammation that helps perpetuate cancer pathology [48]. These antibodies have been demonstrated in hematological neoplasms [36,49], independent of autoimmune diseases and solid tumors such as lung or breast, leaving it unknown if cancer alone could generate an ANCA [48].

### 5.3. ANA

These antibodies tend to be present in some diseases of the connective tissue, healthy individuals, and even patients with chronic infectious diseases [32,40]. These antibodies are directed against the cells’ nuclear components [37,38,39,41] and cytoplasmic components [37].

Patients with positive ANAs have worse progression-free survival and overall survival than those with negative ANAs, especially cancer patients [38]. In patients with hematological neoplasms, such as non-Hodgkin’s lymphoma, and in chronic lymphocytic leukemia (CLL), there is positivity, and it could work as an independent prognostic factor [41,42]. The possibility of using them as prognostic or diagnostic biomarkers turns out to be promising in terms of the detection of a pre-malignant lesion or diagnosis in the early stages [37].

## 6. Mechanisms of Cellular Activation of aPL and Hematological Malignancies

Considering that several signaling mechanisms interact simultaneously in terms of the interaction of aPL with different antigens [50], the interaction of β2-glycoprotein I stands out since changing its structural configuration from closed to open in response to inflammatory changes or the presence of anionic phospholipids allows the interaction of aPL with the protein by exposing the epitope and allowing the coupling of autoantibodies and, consequently, their binding to cellular receptors on the cells of the immune system [24,25,28,29,50]. As for the receptors, there are three candidates, the phosphatidylserine one being of particular interest since it can be externalized only with proinflammatory cytokine stimulation sequenced from a phospholipid activation. Other proposed receptors include annexin A2 and the Toll receptor TLR4 [25].

Often, it can be challenging to elucidate how the aPLs interact or are present in different hematological malignancies, so to explain, we have to address the two different proteins and their actions: annexin A2 (ANX A2) and Toll-like receptor 4 (TLR4) [51,52].

ANX A2 is expressed on the surface of inflammatory and endothelial cells and different types of cancer cells, mediating the communication between the intercellular and extracellular microenviroments; additionally, it participates in the communication related to cell survival. In addition, this protein is involved in cytokinesis, exocytosis, endocytosis, intracellular trafficking cell junctions, and the regulation of mitotic cell division, giving crucial importance to the signaling for cell survival and proliferation as well as to neoangiogenesis, tumor growth, and metastasis [51,53,54,55]. It is linked to tumor progression and even treatment resistance [56].

On the other hand, TLR4 is part of an extensive family of transmembrane receptors that are expressed in the cells that compose the innate immune system, and these receptors are known as pattern recognition receptors for their ability to recognize the pathogen-associated molecular patterns (PAMPs) and damage-associated molecular patterns (DAMPs) [57,58], enabling the activation of innate and adaptative immune responses [57]. Furthermore, the TLRs have a significant role in the signaling pathways for different proinflammatory cytokines [57,58]. Inflammation in cancer facilitates the formation and progression of tumors from mutated cells or cells that are already damaged. This chronic inflammation increases by the enhancement of the microenvironment and high growth and survival factors; Moreover, the molecular signals are mediated by TLR4 and other TLRs [57,59,60].

aPL has reactivity with phospholipid-binding proteins, anionic phospholipids, some proteins, or some phospholipid-protein complexes such as ANXA 2 [61,62]. As we have said before, the protein β2-GPI is the principal trigger in antiphospholipid syndrome (APS), but it can bind and create a complex with ANXA2, possibly being one of the antigens that can trigger APS [63]. ANXA 2 also can be activated by TLR4, which can go through up-regulation or through a mediated signaling on macrophages in inflammatory events [64]. In addition to these findings, in the particular case of anti-B2 GP I, the co-receptor function of TLR4 and ANXA 2 has been reported, giving us a signaling pathway that could be a therapeutic target for several pathologies, among these, APS [65,66].

The mechanism explained before, between TLR4 and ANXA 2 and anti-B2 GP I, could give us an explanation for the pathological activation of the APS [67]. Thrombosis is more frequent in cancer patients than in general, without distinguishing between hematological or solid neoplasms [46]. From the international registry of patients with catastrophic APS, up to 16% of them suffered comorbid neoplasms, without specifying the origin of this [68]. However, there is evidence that the most frequent neoplasms associated with catastrophic APS are hematological neoplasms [69].

Currently, there are some examples of hematological malignancies in conjunction with APS, those being non-Hodgkin lymphoma (NHL) [70], extranodal NHL [71], and NHL [72]. In the last five years, multiple cases of acute and chronic leukemia with the presence of APS have also been documented [73,74] and a case of concomitant multiple myeloma with PAS [75]. Clinically, there are undefined associations with these antibodies’ presence [23], but a connection exists between its prevalence and the occurrence of thrombotic events in cancer patients [22,33].

## 7. Conclusions

The relevance of aPL in cancer has been under study but still lacks enough focus on the implications of patients that undergo hematological malignancies and aPL positives, especially for relationships between these antibodies and the development of prothrombotics states. The emphasis of the study of these antibodies could direct us to better management of patients with hematological neoplasms, taking as a goal the prediction of the prognosis. Having new targets in the treatment could lead us to an enhancement of overall survival and better quality of life.

## Figures and Tables

**Table 1:** Categories and functions of different types of lipids according to LIPID MAPS.

	Categories					
	Fatty and Conjugated Acids	Glycerolipids	Glycerophospholipids	Sterols		
			Sphingolipids	Prenol Lipids	Saccharolipids	Polyketide
Function	Promote angiogenesis, reduce inflammation	Energy store, second messenger signaling lipid, stabilization of T cell activation and proliferation	Structural components of the membrane affects membrane fluidity. Component of the cell membrane induces lipotoxicity and inflammation, regulates apoptosis.	Regulates the progression of aging-related diseases, diabetes	Cell wall lipids from plants, bacteria, and fungi	Anti-microbial, anti-parasitic, and anticancer functions
Examples	Octadecanoids, eicosanoids, docosanoids, fatty acids esters, fatty amides, fatty acid glycosides	Monoradyl-glycerol, diradyl-glycerol, triadyl-glycerol, glycosyl-monoradyl-glycerol and glycosyl-diradyl-glycerol	Sphingoid bases, ceramides, phosphosphingolipids, neutral glycosphingolipids, acid glycosphingolipids.	Isoprenoids, polyprenols	Acrylamine-Sugar	Linear polyketide

Adapted from: Zhang, C.; Wang, K.; Yang, L.; Liu, R.; Chu, Y.; Qin, X.; et al. Lipid metabolism in inflammation-related diseases. *Analyst* **2018**, *143*, 4526–4536. Ref. [6].

**Table 2:** Presence of autoantibodies in hematological neoplasms.

Type of Neoplasm	Type of Autoantibodies	Percentage
Hodgkin lymphoma [22,23,26,43]	LA	67%
	aCL	67%
	anti β2 GP I	46%
Non Hodgkin lymphoma [22,23,26,42,43,44]	ANAs	30.5%
	aCL	22%
	LA	48–61%
	anti β2 GP I	46%
Acute and chronic leukemia [22,23,26,41]	ANAs	
	aCL	40–62%
	LA	48%
	anti β2 GP I	6%
Myeloma multiple [22,23]	aCL	40–62%
	LA	48%

LA: lupus anticoagulant; aCL: anticardiolipin; anti β2 GP I: anti-β2 glycoprotein I; ANAs: antinuclear antibodies.

## Data Availability

Non applicable.

## Abbreviations

ACC	Acetyl-CoA carboxylase
AMPK	AMP-activated kinase
anti-β2GP-I	Anti-β2 glycoprotein I
anti-dsDNA	Double-stranded anti-DNA antibodies
aCL	Anticardiolipin
ADCC	Antibody-dependent cell-mediated cytotoxicity
ANCA	Anti-cytoplasmic neutrophil antibodies
aPL	Antiphospholipid antibodies
APS	Antiphospholipid syndrome
CCL	Chronic lymphocytic leukemia
CCP	Cytidine choline diphosphate
DNA	Deoxyribonucleic acid
ENAs	Autoantibodies against extractable nuclear antigens
FAS	Fatty acid synthase
LA	Lupus anticoagulant
HL	Hodgkin Lymphoma
NETs	Extracellular neutrophil traps
NHL	Non-Hodgkin Lymphoma
PC	Phosphatidylcholine
PEMT	Phosphatidylethanolamine N-methyltransferase
TLR2	Receptor type Toll 2
TLR4	Receptor type Toll 4
TLR7	Receptor type Toll 7
